# Linking Epitope‐Specific T‐Cell Receptors to IFNγ Secretion Using Nanovial Technology

**DOI:** 10.1002/eji.202451666

**Published:** 2025-05-22

**Authors:** Jet van den Dijssel, Tejas Menon, Hayley A. McQuilten, Oliver Eltherington, Alexis Gonzalez, Sevana Baghdasarian, Citradewi Soemardy, Dino Di Carlo, Klaas P.J.M. van Gisbergen, Anja ten Brinke, Carolien E. van de Sandt

**Affiliations:** ^1^ Sanquin Research and Landsteiner Laboratory, Amsterdam UMC University of Amsterdam Amsterdam The Netherlands; ^2^ Amsterdam Institute for Immunology and Infectious Diseases Amsterdam The Netherlands; ^3^ Department of Microbiology and Immunology University of Melbourne at the Peter Doherty Institute for Infection and Immunity Melbourne Australia; ^4^ Melbourne Cytometry Platform University of Melbourne Melbourne Australia; ^5^ Department of Chemical and Biomolecular Engineering University of California Los Angeles Los Angeles California USA; ^6^ Department of Bioengineering University of California Los Angeles Los Angeles California USA; ^7^ Partillion Bioscience Pasadena California USA; ^8^ Department of Mechanical and Aerospace Engineering University of California Los Angeles Los Angeles California USA; ^9^ California NanoSystems Institute (CNSI) University of California Los Angeles Los Angeles California USA; ^10^ Physiology and Cancer Programme, Champalimaud Research Champalimaud Foundation Lisbon Portugal

**Keywords:** cytokine secretion, epitope‐specific CD8^+^ T‐cells, nanovials, T‐cell receptor

## Abstract

To link IFNγ‐secretion levels of epitope‐specific T‐cells with their TCRαβ, we coated nanovials with pHLA‐I to capture and activate epitope‐specific T‐cells and their secreted IFNγ, followed by index‐sorting and TCRαβ sequencing. We demonstrate that nanovials are a promising tool to link single epitope‐specific TCRαβ clonotypes to the cell's functional properties.

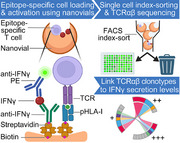

**Abbreviations**
A2: HLA‐A*02:01
CDR: complementarity‐determining region
ICS: intracellular cytokine staining
IFNγ: interferon‐gamma
IL‐2: interleukin‐2
pHLA‐I: peptide bound to human leukocyte antigen class‐I
TCR: T‐cell receptor
TNF: tumor necrosis factor

CD8^+^ T‐cells play a key role in controlling viral infections by eliminating virus‐infected cells. Their T‐cell receptor (TCR) recognizes epitopes (peptides bound to HLA class‐I (pHLA‐I)) on the infected cell surface. This results in CD8^+^ T‐cell activation, including cytokine secretion, like interferon‐gamma (IFNγ), differentiation, and memory formation [1, 2]. TCRs affect the functionality, phenotype, and secretory function of epitope‐specific CD8^+^ T‐cells [1, 3, 4]. However, linking TCR clonotypes directly to functionality, as determined by cytokine secretion, remains technically challenging.

Cytokine production upon epitope‐specific CD8^+^ T‐cell activation can be assessed by combining pHLA‐tetramers with intracellular cytokine staining (ICS) [1, 5]. However, the required fixation and permeabilization degrades mRNA, making additional paired‐TCRαβ analysis challenging. Some studies successfully sequenced cytokine mRNA after ICS; however, its suitability for sensitive paired‐TCRαβ sequencing remains unclear [6]. Single‐cell transcriptomics links TCRs with cytokine mRNA levels, but mRNA levels poorly correlate with secreted cytokine levels [7]. The cytokine‐catch assay utilizes antibodies that simultaneously bind unfixed T‐cell and secreted cytokines, making it compatible with the TCRαβ‐multiplex. However, secreted cytokines can be captured by antibodies on noncytokine‐producing cells resulting in false positives [8]. Therefore, new technologies are required to unequivocally link cytokine‐secretion levels to their TCR.

Nanovial technology uses cavity‐containing hydrogel microparticles to capture cells and their secreted cytokines [9, 10]. The nanovial cavity is coated with antibodies that bind molecules expressed on the surface of cells of interest. Nanovials can also be coated by pHLA‐I complexes (monomers) which bind TCRs and simultaneously activate cells in an epitope‐specific manner (Figure 1A). Additional anti‐cytokine antibodies also coat the nanovial‐cavity to catch secreted cytokines. Their cup‐like structure prevents spillover of secreted cytokines to neighboring cells [9, 10]. Following incubation to allow cytokine secretion, nanovials are stained using Calcein‐AM (a live‐cell stain to separate empty from cell‐loaded nanovials), and fluorescent antibodies to detect the cytokine and cell surface markers. Nanovials containing cytokine‐secreting cells can be (single‐cell) sorted, without fixation or permeabilization, allowing further cell culture or adoptive transfer [10]. Importantly, cells can be used for TCRαβ or scRNA sequencing, to link cytokine‐secretion levels with TCR or gene‐expression profiles. However, it remains unclear how nanovial technology compares to conventional ICS.

**FIGURE 1:**
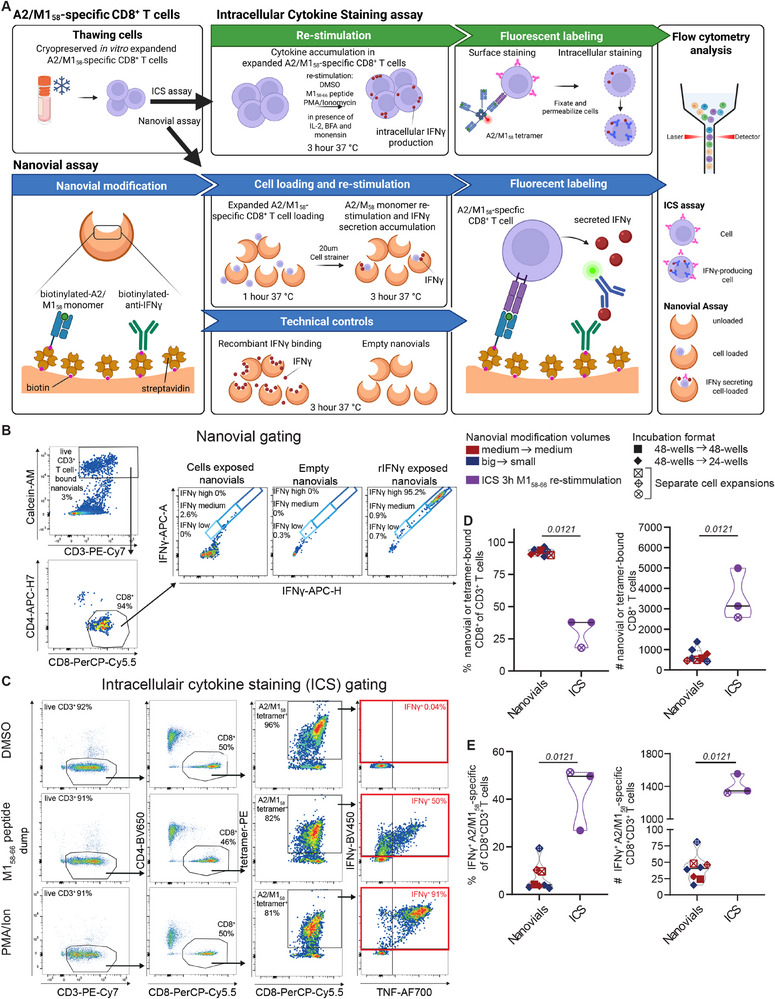
Nanovial‐captured and activated A2/M1_58_‐specific CD8^+^ T‐cells secrete IFNγ. (A) Workflow of the ICS and nanovial assays; created with BioRender. Representative gating strategy of the (B) nanovial assay and (C) ICS, full gating strategy in Figure S1. Frequency and number of (D) nanovial or tetramer‐bound CD8^+^ T‐cells or (E) IFNγ‐secreting (nanovial) and IFNγ‐producing (ICS) A2/M1_58_‐specific CD8^+^ T‐cells. Nanovials *n* = 8, ICS *n* = 3 conditions, measured across 5 and 2 experiments respectively. Similar conditions were measured across independent experiments. Horizontal lines indicate the median. Statistical analysis by the unpaired Mann–Whitney *U*‐test. Significant *p*‐values are displayed above the graphs.

Nanovials were coated with anti‐IFNγ antibodies and the prominent and high‐affinity influenza HLA‐A*02:01‐M1_58‐66_ (A2/M1_58_) epitope [1, 11, 12] to capture and activate A2/M1_58_‐specific CD8^+^ T‐cells. Modified nanovials were incubated with in vitro A2/M1_58_‐expanded CD8^+^ T‐cells in a 1.6 to 1 ratio (64,000 nanovials to 40,000 cells) (Figure 1A). Modified nanovials incubated with recombinant IFNγ (rIFNγ) and empty nanovials served as positive and negative controls, respectively (Figure 1A,B; Figure S1A). Different conditions, including nanovial modification volumes, cell incubation formats, and incubation times were assessed to optimize epitope‐specific CD8^+^ T‐cell‐nanovial binding (Figure S2A). A 3 h incubation resulted in significantly higher CD8^+^ T‐cell‐loaded nanovial numbers compared with a 5 h incubation (Figure S2B), with significantly more medium‐ and high‐secreting cells (Figure S2C). This suggests that A2/M1_58_‐specific CD8^+^ T‐cells might dissociate from nanovials over time. Other optimizations did not considerably impact T‐cell loading. Concurrently, expanded A2/M1_58_‐specific CD8^+^ T‐cells were re‐stimulated with M1_58‐66_ peptide, PMA/Ionomycin (positive control), or DMSO (negative control) to access IFNγ‐production by ICS (Figure 1A,C; Figure S1B). IFNγ^+^A2/M1_58_^+^CD8^+^ T‐cell frequencies were similar for 3 and 5 h peptide restimulation (Figure S2D). Therefore, IFNγ‐production by A2/M1_58_‐specific CD8^+^ T‐cells was compared between nanovials and ICS at 3 h.

Ninety‐two percent of live CD3^+^ T‐cells loaded on A2/M1_58_‐coated nanovials (median) were CD8^+^ T‐cells, which was significantly higher than the median 38% A2/M1_58_ tetramer^+^CD8^+^ T‐cells in the ICS (% of live CD3^+^ T‐cells) (Figure 1D). However, a significantly higher number of A2/M1_58_‐tetramer ^+^CD8^+^ T‐cells was detected in the ICS (median 3136 cells) compared with nanovial‐bound CD8^+^ T‐cells (median 575 cells). This indicates that although pHLA‐coated nanovials bind cells in a highly specific manner, not all epitope‐specific cells are captured from the target population (Figure 1D). This observation was supported by significantly higher frequency and number of IFNγ‐producing A2/M1_58_^+^CD8^+^ T‐cells in the ICS (median 50%, 1344 cells) versus nanovial‐bound IFNγ‐secreting A2/M1_58_^+^CD8^+^ T‐cells (median 4%, 41 cells) (Figure 1E).

To confirm that low cell‐nanovial binding did not result from inefficient nanovial‐coating with biotinylated A2/M1_58_‐monomers and anti‐IFNγ, empty modified nanovials were stained with anti‐HLA‐A2‐FITC and anti‐IFNγ‐APC after incubation with rIFNγ. Our findings confirmed that nanovials were adequately coated with A2/M1_58_ monomers and anti‐IFNγ capture reagents (Figure S2E). However, further optimization is required to enhance the capture of IFNγ‐secreting epitope‐specific CD8^+^ T‐cells, to replicate the binding efficiency observed in earlier studies [10]. Differences in IFNγ‐production through intracellular accumulation (ICS) or secretion (nanovials) may be attributed to reduced numbers of IFNγ‐secreting cells identified in the nanovial assay. Conversely, activated T‐cells may decrease their TCR expression, resulting in reduced avidity for the monomer‐coated nanovials. Indeed, tetramer staining of M1_58–66_‐peptide and PMA/ionomycin stimulated cells in the ICS was less bright compared with the DMSO control (Figure 1C; Figure S2F). Co‐coating nanovials with additional capture reagents for molecules upregulated on activated T‐cells, like ICAM1, may increase the avidity of activated T‐cells [13].

Next, as proof‐of‐principle, nanovial‐bound IFNγ‐secreting A2/M1_58_‐specific CD8^+^ T‐cells were index‐sorted followed by paired‐TCRαβ sequencing to link their paired‐TCRαβ and IFNγ‐secretion levels. Since ICS and TCRαβ‐multiplex sequencing are incompatible, we compared TCR repertoires of index‐sorted nanovial‐bound IFNγ‐secreting CD8^+^ T‐cells and A2/M1_58_‐tetramer^+^CD8^+^ T‐cells (Figure S3A,B). Nanovial‐bound cells were sequenced using our standard TCRαβ‐multiplex protocol [1]. TCRs consist of an alpha (α) and beta (β) chain, composed of a variable, junctional, and constant region. Their hypervariable complementarity‐determining region 3 (CDR3) determines the TCR specificity [14]. Circos analysis of TCRα and TCRβ variable regions (TRAV and TRBV), revealed that nanovial‐ and tetramer‐bound A2/M1_58_‐specific CD8^+^ T‐cells shared 5/12 TRAV genes (including TRAV27) and were both dominated by TRBV19‐expressing clonotypes (Figure S3C, Table S1). Furthermore, CDR3‐motifs overlapped between nanovial‐ and tetramer‐bound A2/M1_58_‐specific CD8^+^ T‐cells (Figure S3D). Importantly, TRAV27, TRBV19, and the CDR3β‐(I)RS‐motif are characteristic of high‐affinity A2/M1_58_‐specific clonotypes [1, 12, 15], confirming that nanovial‐bound CD8^+^ T‐cells were A2/M1_58_‐specific. Overall, four paired‐TCRαβ clonotypes were shared between nanovial‐ and tetramer‐bound A2/M1_58_‐specific CD8^+^ T‐cells (Figure S3E). To link paired‐TCRαβ clonotypes with their IFNγ‐secretion level, index‐sorted nanovial‐bound A2/M1_58_‐specific CD8^+^ T‐cells were divided into low, medium, and high IFNγ‐secreting populations. The “bottom” gate contains cells with anti‐IFNγ stained cell surfaces rather than secreted IFNγ [10] (Figure S3B). Paired‐TCRαβ clonotypes were shared between IFNγ‐secreting populations, but only one clonotype was detected among all IFNγ populations (Figure S3F–G; clone‐ID “A”, Table S1).

Overall, nanovials are a promising tool to link cytokine secretion by epitope‐specific CD8^+^ T‐cells to their paired TCRαβ. However, additional optimization is needed to further improve the capture and activation of epitope‐specific CD8^+^ T‐cells. Although we identified TCR overlap between nanovial‐ and tetramer‐bound A2/M1_58_‐specific CD8^+^ T‐cell populations, larger studies are needed to conclusively link specific TCRαβ‐features to a specific IFNγ‐secretion level. Importantly, the nanovial assay can be extended to capture additional secreted cytokines, including TNF and IL‐2, to study the polyfunctionality of epitope‐specific T‐cells [10], while staining of additional surface markers allows further in‐depth phenotypic characterization of cytokine‐secreting cells.

## Conflicts of Interest

D. D. C. and the Regents of the University of California have financial interests in Partillion Bioscience Corporation, which sells nanovials. H. A. M. is a consultant for Ena Respiratory. The remaining authors declare no conflicts of interest.

## Supporting information



Supporting Information

Supporting Information

Supporting Information

## Data Availability

TCR data are included in Table S1. Source data are deposited in Mendeley [DOI:10.17632/ptnrwb9kyk.1]. Additional data are available from the corresponding author upon reasonable request.
