# Supplementary material for: Linking Epitope‐Specific T‐Cell Receptors to IFNγ Secretion Using Nanovial Technology
Source: Eur J Immunol. 2025 May 22;55(5):e202451666. doi: 10.1002/eji.202451666 (PMC12099177; doi:10.1002/eji.202451666)
Supplement: Supplementary file 1 — Supporting Information [file EJI-55-e202451666-s002.docx]

# Supporting information

### Methods

### Study participant and ethics

Peripheral blood of one HLA-A*02:01 expressing healthy female adult donor was collected in heparinised tubes from which peripheral blood mononuclear cells (PBMCs) were isolated using Ficoll-Paque (GE HealthCare) gradient centrifugation and cryopreserved in liquid nitrogen until required. HLA class I molecular genotyping was performed from genomic DNA by the Australian Red Cross Lifeblood.

The donor was recruited via the University of Melbourne, provided informed written consent and did not receive any compensation. Experiments were conformed to the Declaration of Helsinki Principles and the Australian National Health and Medical Research Council Code of Practice. The study was approved by the Human Research Ethics Committee of the University of Melbourne (Ethics ID #13344).

### Peptides and tetramers

The influenza A M1_58–66_ peptide (GILGFVFTL) was purchased from GeneScript. HLA-A*02:01/M1_58-66_ biotinylated monomers were provided by Prof Andrew Brooks and Jie Lin (Department of Microbiology and Immunology, University of Melbourne, Melbourne, Australia). The monomers were conjugated 8:1 to streptavidin-PE (BD Pharmingen, cat 554061) to generate A2/M1_58_-PE tetramers.

### A2/M1_58_-specific CD8^+^ T-cell expansion

PBMCs were thawed in complete RPMI (cRPMI) medium (RPMI1640 medium (Invitrogen), 1 mM MEM sodium pyruvate (Gibco), supplemented with 2 mM l-glutamine (Gibco), 5 mM HEPES buffer solution (Gibco), 100 µM MEM non-essential amino acids (Gibco), 55 µM 2-mercaptoethanol (Gibco), 100 µg/ml streptomycin (Gibco), 100 U/ml penicillin (Gibco) and 10% fetal bovine serum (Gibco)) supplemented with 2 µg/ml deoxyribonuclease I (DNase; Sigma-Aldrich) and washed once with serum-free RPMI. A2/M1_58_-specific CD8^+^ T-cells were amplified as described previously [1], briefly autologous PBMCs were pulsed with 10 μM M1_58-66_ peptide in serum-free RPMI for 60 min at 37°C and subsequently washed with RPMI. Peptide-pulsed PBMCs were incubated with autologous nonpeptide-pulsed PBMCs and cultured for 12 days in cRPMI. Cultures were supplemented with 20 U/ml recombinant IL-2 (Roche) on day 4 and with fresh media every 2–4 days, after which cells were cryopreserved in liquid nitrogen until required.

### Intra-cellular staining

Expanded A2/M1_58_-specific CD8^+^ T-cells were thawed in cRPMI supplemented with 2 µg/ml deoxyribonuclease I (DNase; Sigma-Aldrich) and washed once with cRPMI. Per condition, 40,000 cells were re-stimulated with M1_58-66_ peptide (1 µM), PMA/Ionomycin (5 ng/ml PMA and 1 µg/ml ionomycin) (positive control), or DMSO (negative control), and cultured for 3 or 5 h in the presence of Brefeldin A (Golgi Plug, BD bioscience), Monensin (Golgi Stop, BD bioscience) and 10 U/ml recombinant IL-2 at 37 °C (5% CO_2_). After re-stimulation, cells were surface stained on ice for 30 min with Live/Dead near-infrared (NIR) Invitrogen cat. L34976, 1:800), anti-CD19-APC-H7 (clone SJ25C1, BD Pharmingen cat. 560177, 1:100), CD14-APC-H7 (clone MφP9, BD Pharmingen cat. 560180, 1:100), anti-CD3-PE-Cy7 (clone UCHT1, BD Pharmingen cat. 563423, 1:50), anti-CD8-PerCP-Cy5.5 (clone SK1, BD Pharmingen cat. 565310, 1:50), anti-CD4-BV650 (clone SK3, BD Horizon cat. 563875, 1:100) and PE conjugated A2/M1_58_ tetramers. After washing cells were fixed with BD Fix-Perm buffer (BD biosciences) for 20 min, followed by intracellular staining on ice for 30 min with anti-TNF-AF700 (clone MAb11, BD Pharmingen cat. 557996, 1:50) and anti-IFN-γ-V450 (clone B27, BD Pharmingen cat. 560371, 1:100) in perm wash buffer (BD Biosciences). Cells were washed, resuspended in MACS buffer (PBS plus 0.5% BSA and 2 mM EDTA), acquired on the BD Fortessa (BD Biosciences) and analysed using Flowjo (V10.8.1, BD bioscience).

### Nanovial modification

Sterile 35 μm biotin-coated nanovials (Partillion Bioscience, Human IFNg secretion assay kit APC, cat. CK-103-A) were modified per manufacturer protocol (protocol: Nanovial IFN-γ Secretion Assay Kit (PE), Rev. Date: June 2023), with the following adjustments: Nanovials were washed with the Partillion wash buffer and incubated with streptavidin (Streptavidin from the Partillion kit scaled to number of nanovials used or 300 μg/ml of streptavidin from Thermo Fisher cat. 43-4302) on a rotator for 30 minutes at room temperature (RT), after which they were washed and incubated with the capture mix containing 25 µg A2/M1_58_ biotinylated monomer per million nanovials and anti-IFNγ (either anti-IFNγ from the Partillion kit scaled to number of nanovials used or 40 μg/ml from R&D systems, BAF285) on a rotator for 30 minutes at RT. The streptavidin and capture mix incubation volumes were executed in either 200 μl (big volume), 1 ml per million nanovials (medium volume, 70-90 μl) or by spiking the reagents into the nanovial pellet (small volume). Nanovials were stored overnight at 4°C and washed twice before incubating with cells.

### Nanovial cell loading, IFNγ capture and staining

A2/M1_58_ monomer and anti-IFNγ modified nanovials were either incubated in cRPMI with thawed expanded A2/M1_58_-specific CD8^+^ T-cells, 100 ng/mL human recombinant IFNγ (rIFNγ, rIFNγ, Biolegend cat. 570202) or kept empty. The nanovial modifications, cell loading and staining were performed according to the manufacturer´s instructions, while including variations of the protocol in an attempt to further optimize the binding capacity of the nanovials. Briefly, 64,000 modified nanovials were suspended in 200µl of cRPMI containing 40,000 M1_58_ expanded PBMCs (ratio 1.6 nanovials per cell). The cell-nanovial mix was incubated in cRPMI for 1 hour at 37 °C in either a 1.5 mL Eppendorf tube, 96-well flat-bottom plate well or 48-well flat-bottom plate well. After 30 min the suspension of nanovials and cells was remixed by carefully pipetting up and down 5 times. To remove unbound cells, nanovials were strained using a 20μm cell strainer (pluriStrainer Mini, cat. 43-10020-40) after 1 hour. Nanovials (with or without loaded cells) were recovered into a 10 ml tube by inverting and flushing the cell strainer with wash buffer (Partillion kit). The nanovials were centrifuged at 200g for 5 minutes, resuspended in 200 or 600µl cRPMI and incubated for 3 or 5 hours at 37 °C in a 1.5 mL Eppendorf tube (200µl), 96-well flat-bottom (200µl), 48-well flat-bottom (200µl) or 24-well flat-bottom plate (600µl). Modified nanovial controls were incubated with 100 ng/mL rIFNγ in cRPMI (positive control, Biolegend cat. 570202) or cRPMI only (negative control and A2-FITC staining). FACS single-cell index-sorting was performed by incubating 64,000 nanovials resuspended in 200µl of cRPMI 48,120 A2/M1_58_ expanded CD8^+^ T-cells (1.33 nanovials per cell).

After incubation nanovials were collected in 1.5 mL Eppendorf tubes and washed twice with staining buffer (PBS with 1% BSA), nanovials were stained for 30 minutes in the fridge (4 °C) with Calcein-AM (BD 564061, 1:2000), anti-CD3-PE-Cy7 (clone UCHT1, BD Pharmingen cat. 563423, 1:50), anti-CD8-PerCP-Cy5.5 (clone SK1, BD Pharmingen cat. 565310, 1:50), anti-CD4-APC-H7 (clone RPA-T4, BD Horizon cat. 560158, 1:50) and anti-IFNγ-APC (Partillion Bioscience kit or Biolegend clone 4S.B3 cat. 502512 or Partillion kit, 1:20). Half of the empty nanovials were stained with anti-Human HLA-A2 FITC (clone BB7.2, BD bioscience cat. 51285, 1:100) in cRPMI while the remaining empty nanovials and rIFNγ nanovials were stained with anti-IFNγ-APC. Nanovials were washed twice in washing buffer, resuspended in wash buffer and acquired on the BD Fortessa (BD Biosciences) or single-cell index-sorted on the BD FACSAria III (BD Biosciences). All samples were analysed using Flowjo (V10.8.1, BD bioscience).

### Tetramer staining

Expanded A2/M1_58_-specific CD8^+^ T-cells (1 million cells) were thawed and rested for 4h in a 48-well plate in 800µl cRPMI at 37°C. After incubation the expanded A2/M1_58_-specific CD8^+^ T-cells were washed with MACS buffer and the surface was stained for 30 min on ice with Live/Dead near-infrared (NIR) (Invitrogen cat. L34976, 1:800), anti-CD19-APC-H7 (clone SJ25C1, BD Pharmingen cat. 560177, 1:100), CD14-APC-H7 (clone MφP9, BD Pharmingen cat. 560180, 1:100), anti-CD3-BV510 (clone OKT3, Biolegend cat. 317332, 1:200), anti-CD8-PerCP-Cy5.5 (clone SK1, BD Pharmingen cat. 565310, 1:50), anti-CD4-BV650 (clone SK3, BD Horizon cat. 563875, 1:100), anti-CD27-BV711 (clone L128, BD Horizon cat. 563167, 1:200), anti-CD45RA-FITC (clone HI100, BD Horizon cat. 555488, 1:200), anti-CD95-PE-CF595 (clone DX2, BD Horizon cat. 562395, 1:100) and PE-conjugated A2/M1_58_ tetramers in MACS. Cells were washed twice and resuspended in MACS buffer and single-cell index-sorted using the BD FACSAria III (BD Biosciences), followed by analysis using Flowjo (V10.8.1, BD bioscience).

### FACS sorting

FACS sorting protocol was adapted from *De Rutte et al.* [2]. In short, single-cell index sorting was performed using a 100µm nozzle on a BD FACS™ Aria III cell sorter (BD Biosciences) at low volumetric speed and event rate. The drop charge delay (DCD) for tetramer-stained cells was set using BD FACS™ Accudrop beads following the manufacturer’s recommended procedure. The optimal DCD for the nanovials was identified using 32 µm Sphero™ AccuCount Ultra-Rainbow particles (Spherotech, Cat. No. ACURFP2.5-300-5) ran through the auto drop-delay programme on DIVA. This was verified by triplicate test sorts of 50 empty nanovials onto a slide and manual counting using a light microscope. The nanovial sort DCD value was found to be 0.76 drop equivalent (or 24.6 µs) over from the Accudrop beads-defined optimum. These settings improved recovery of empty nanovials from 7% (Accudrop beads) to 87% (32 µm beads). Nanovial-bound IFNγ^+^CD8^+^ T-cells and A2/M1_58_ tetramer^+^CD8^+^ T-cells were single-cell index-sorted into a chilled 96-well twin.tec PCR plates (Eppendorf) and immediately stored at −80 °C until required.

### Single-cell RT-PCR and paired TCRαβ sequencing

Single-cell paired CDR3α and CDR3β regions were analysed by multiplex-nested PCR with reverse transcription followed by sequencing of the CDRα and CDRβ products, as described previously [1],[3]. Sequences were examined with FinchTV and VJ regions were identified using IMGT (www.imgt.org/IMGT_vquest). TCR sequences were parsed using the TCRdist analytical pipeline [4]. Clonotypes were defined as single-cell TCRαβ pairs that exhibit the same V, J and CDR3 regions. The motifs were computed by the TCRdist algorithm [4] and are reflective of CDR3 sequences of variable lengths, motif scores were determined by chi-squared, with values above 90 considered significant. Circos plots were created using the circlize package [5] in R v4.2.2 (The Comprehensive R Archive Network (CRAN)).

### Statistics

Statistical analysis was performed in GraphPad Prism (v.9.1.1, GraphPad). A two-sided Mann-Whitney U-test was used when comparing two groups. The Kruskal-Wallis Test followed by Dunn’s multiple comparison Test was performed to study the differences between DMSO and M1_58_ peptide-stimulated or DMSO and PMA/Ionomycin-stimulated cells. Differences were considered significant at *p* ≤ 0.05.

### Data availability

TCR data is included in Supplementary Table 1. Source data are deposited in Mendeley [DOI:10.17632/ptnrwb9kyk.1]. Additional data are available from the corresponding author upon reasonable request.

### Authors contributions

CES conceptualised, led and supervised the study. JD and CES designed the experiments. JD, OE, AG and CES performed the experiments. JD, TM and CES analysed the data. TM and HAM supplied R scripts for visualization. CES recruited the donor. JD, OE, AG, SB, CS, DDC, KPJMG, AB and CES provided intellectual input into the experimental design. AB and CES provided funding. JD and CES wrote the manuscript. All authors reviewed and approved the manuscript.

### References

1. van de Sandt CE, Clemens EB, Grant EJ, Rowntree LC, Sant S, Halim H, Crowe J, et al. Challenging immunodominance of influenza-specific CD8+ T cell responses restricted by the risk-associated HLA-A*68:01 allomorph. Nat Commun. 2019; 10:5579.
2. De Rutte J, Dimatteo R, Zhu S, Archang MM, Carlo D Di. Sorting single-cell microcarriers using commercial flow cytometers HHS Public Access. SLAS Technol. 2022; 27:150–159.
3. Wang GC, Dash P, McCullers JA, Doherty PC, Thomas PG. T-cell receptor αβ diversity inversely correlates with pathogen-specific antibody levels in human cytomegalovirus infection. Sci Transl Med. 2012; 4:128ra42.
4. Dash P, Fiore-Gartland AJ, Hertz T, Wang GC, Sharma S, Souquette A, Crawford JC, et al. Quantifiable predictive features define epitope-specific T cell receptor repertoires. Nature. 2017; 547:89–93.
5. Gu Z, Gu L, Eils R, Schlesner M, Brors B. circlize implements and enhances circular visualization in R. Bioinformatics. 2014; 30:2811–2812.
