# Supplementary material for: Linking Epitope‐Specific T‐Cell Receptors to IFNγ Secretion Using Nanovial Technology
Source: Eur J Immunol. 2025 May 22;55(5):e202451666. doi: 10.1002/eji.202451666 (PMC12099177; doi:10.1002/eji.202451666)
Supplement: Supplementary file 2 — Supporting Information [file EJI-55-e202451666-s001.pdf]

A Nanovial assay gating strategy

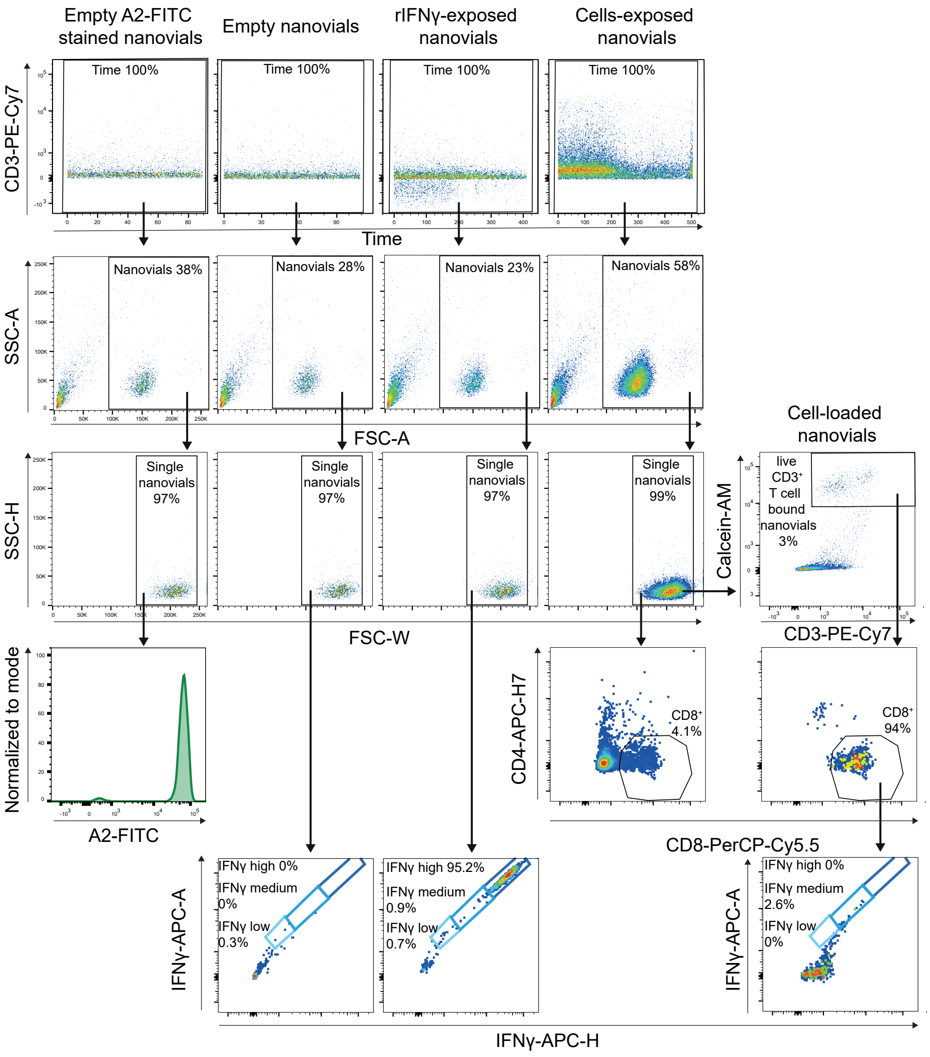

B ICS assay gating strategy

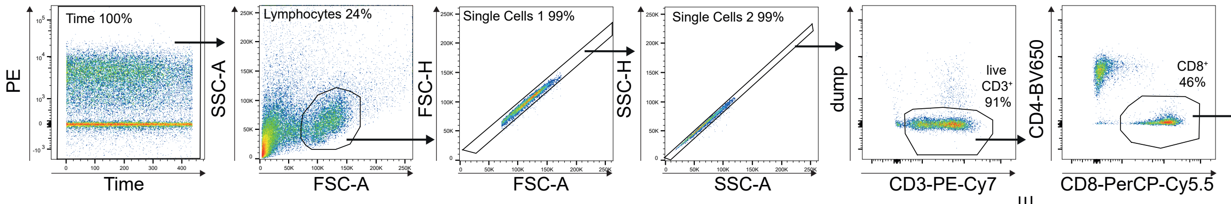

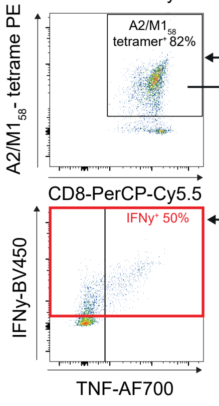

Supplementary Figure 1. Gating strategy IFNγ+A2/M1$_{58}$-specific CD8+ T-cells.

(A) Representative gating strategy to identify IFNγ-secreting A2/M1$_{58}$-specific CD8+ T-cells in nanovial assay. The high, medium and low IFNγ gates were set based on empty and rIFNγ-bound nanovials, while the CD8+ gating of live CD3+ nanovials was based on the total nanovial population. (B) Representative gating strategy to identify IFNγ-producing A2/M1$_{58}$-specific CD8+ T-cells ICS assay. IFNγ+ gating was set based on an unstimulated DMSO sample.

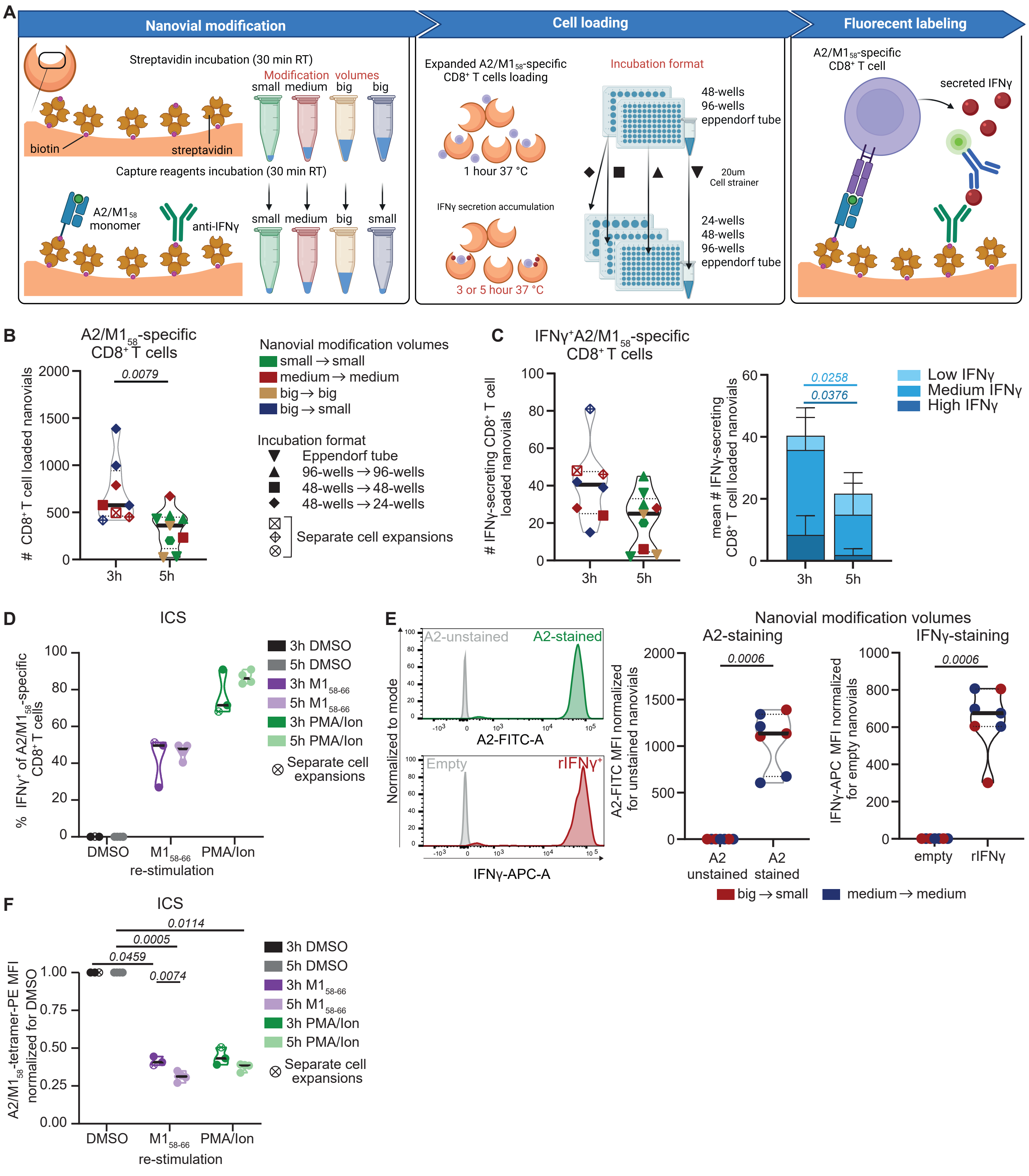
A
Nanovial modification
Cell loading
Fluorecent labeling
Streptavidin incubation (30 min RT)
Modification volumes
small medium big big
biotin
streptavidin
Capture reagents incubation (30 min RT)
A2/M1 58 monomer
anti-IFNγ
small medium big small
Expanded A2/M1 58-specific CD8+ T cells loading
1 hour 37 °C
IFNγ secretion accumulation
3 or 5 hour 37 °C
Incubation format
48-wells
96-wells
eppendorf tube
20um Cell strainer
24-wells
48-wells
96-wells
eppendorf tube
A2/M1 58-specific CD8+ T cell
secreted IFNγ
B
A2/M1 58-specific CD8+ T cells
CD8+ T cell loaded nanovials
0.0079
3h
5h
Nanovial modification volumes
small → small
medium → medium
big → big
big → small
Incubation format
Eppendorf tube
96-wells → 96-wells
48-wells → 48-wells
48-wells → 24-wells
Separate cell expansions
C
IFNγ+A2/M1 58-specific CD8+ T cells
IFNγ-secreting CD8+ T cell loaded nanovials
mean # IFNγ-secreting CD8+ T cell loaded nanovials
0.0258
0.0376
Low IFNγ
Medium IFNγ
High IFNγ
D
ICS
% IFNγ+ of A2/M1 58-specific CD8+ T cells
3h DMSO
5h DMSO
3h M1 58-66
5h M1 58-66
3h PMA/Ion
5h PMA/Ion
Separate cell expansions
DMSO
M1 58-66
PMA/Ion
re-stimulation
E
A2-unstained
A2-stained
Normalized to mode
A2-FITC-A
Empty
rIFNγ+
IFNγ-APC-A
Nanovial modification volumes
A2-staining
IFNγ-staining
A2-FITC MFI normalized for unstained nanovials
0.0006
A2 unstained
A2 stained
IFNγ-APC MFI normalized for empty nanovials
0.0006
empty
rIFNγ
big → small
medium → medium
F
ICS
A2/M1 58-tetramer-PE MFI normalized for DMSO
0.0114
0.0005
0.0459
0.0074
3h DMSO
5h DMSO
3h M1 58-66
5h M1 58-66
3h PMA/Ion
5h PMA/Ion
Separate cell expansions
DMSO
M1 58-66
PMA/Ion
re-stimulation

Supplementary Figure 2. Optimizing cell-loading into nanovials.

(A) Nanovial assay workflow optimization, created with BioRender. Red font indicates steps where varying conditions were tested. (B) Number CD8$^+$ T-cell loaded nanovials and (C) number of IFNγ-secreting CD8$^+$ T-cells-loaded nanovials (left) at different levels of IFNγ-secretion (right) restimulated for 3 or 5-hour (n = 8 at 3h and n = 9 at 5h, measured across 5 and 4 independent experiments respectively). (D) Frequency of IFNγ$^+$A2/M1$_{58}$-tetramer$^+$ CD8$^+$T-cells in ICS assay after 3 or 5-hour re-stimulation with the M1$_{58\text{-}66}$-peptide, PMA-ionomycin (positive control) or DMSO (negative control) (n = 3 at 3h or n = 4 at 5h, measured across 2 or 4 experiments respectively). (E) A2-FITC and IFNγ-APC mean fluorescent intensity (MFI) normalized for negative empty/A2-unstained controls, including representative histograms (n = 7, measured across 6 experiments). (F) DMSO normalized MFI of A2/M1$_{58}$-tetramer-PE stain after 3 or 5-hour re-stimulation with M1$_{58\text{-}66}$-peptide, PMA-ionomycin or DMSO (n = 3 at 3h or n = 4 at 5h, measured across 2 or 4 experiments respectively). (B, C, E) Statistical analysis was performed using the unpaired Mann-Whitney U-test. (F) The Mann-Whitney U-test was performed to compare time points within each condition and the difference between DMSO and M1$_{58\text{-}66}$ or PMA re-stimulation was compared at each time point with the Kruskal-Wallis test with Dunn’s multiple comparison correction. Horizontal lines indicate the median. Significant exact p-values are indicated above the graphs.

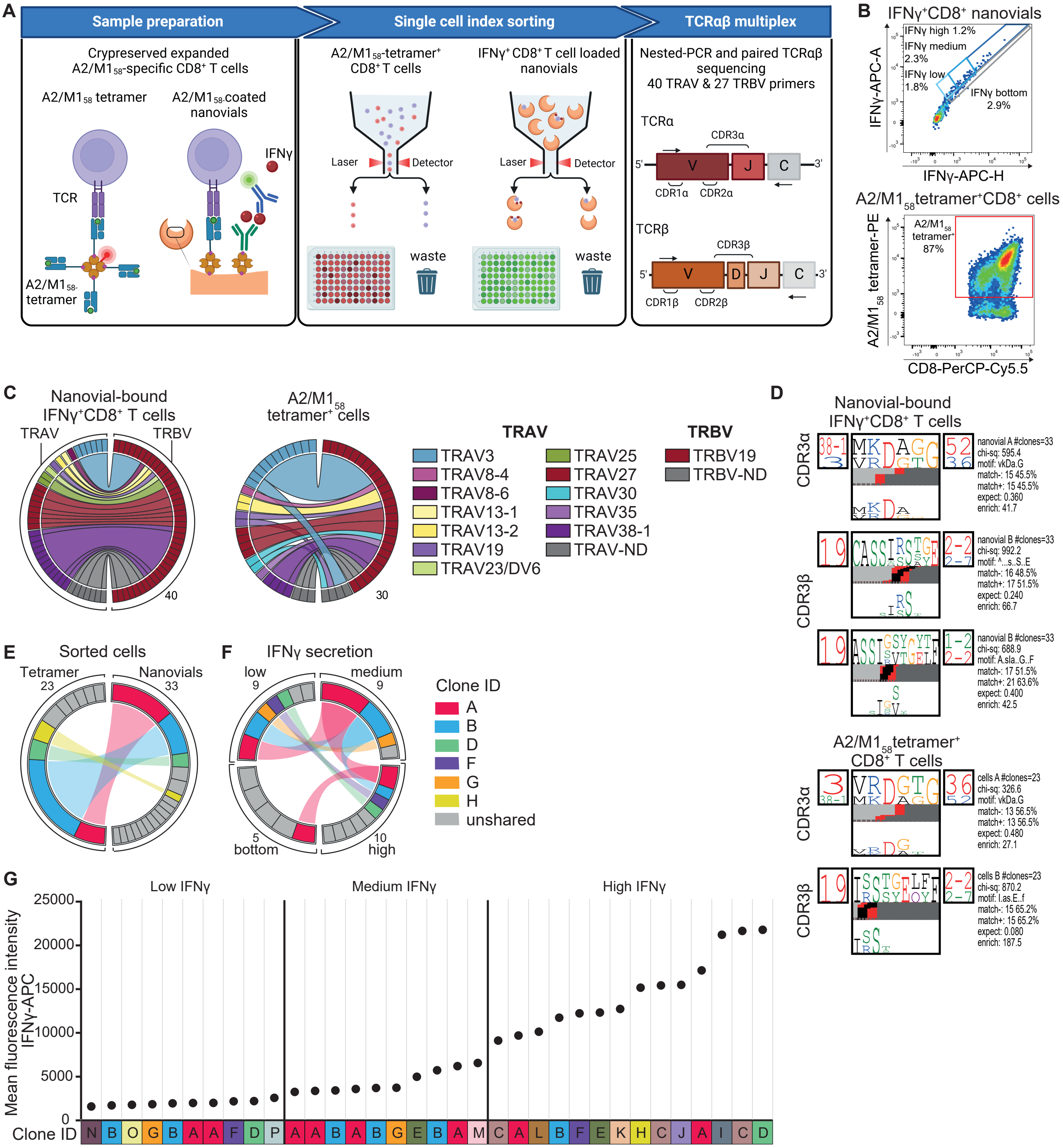
A
Sample preparation
Single cell index sorting
TCRαβ multiplex
Crypreserved expanded A2/M1₅₈-specific CD8⁺ T cells
A2/M1₅₈ tetramer
A2/M1₅₈-coated nanovials
TCR
IFNγ
A2/M1₅₈-tetramer
A2/M1₅₈-tetramer⁺ CD8⁺ T cells
IFNγ⁺ CD8⁺ T cell loaded nanovials
Laser
Detector
waste
Nested-PCR and paired TCRαβ sequencing
40 TRAV & 27 TRBV primers
TCRα
CDR3α
V
J
C
5'
3'
CDR1α
CDR2α
TCRβ
CDR3β
D
CDR1β
CDR2β
B
IFNγ⁺CD8⁺ nanovials
IFNγ high 1.2%
IFNγ medium 2.3%
IFNγ low 1.8%
IFNγ bottom 2.9%
IFNγ-APC-A
IFNγ-APC-H
A2/M1₅₈tetramer⁺CD8⁺ cells
A2/M1₅₈ tetramer⁺ 87%
A2/M1₅₈ tetramer-PE
CD8-PerCP-Cy5.5
C
Nanovial-bound IFNγ⁺CD8⁺ T cells
TRAV
TRBV
40
A2/M1₅₈ tetramer⁺ cells
30
TRAV
TRAV3
TRAV8-4
TRAV8-6
TRAV13-1
TRAV13-2
TRAV19
TRAV23/DV6
TRAV25
TRAV27
TRAV30
TRAV35
TRAV38-1
TRAV-ND
TRBV
TRBV19
TRBV-ND
D
Nanovial-bound IFNγ⁺CD8⁺ T cells
CDR3α
nanovial A #clones=33
chi-sq: 595.4
motif: vkDa.G
match-: 15 45.5%
match+: 15 45.5%
expect: 0.360
enrich: 41.7
CDR3β
nanovial B #clones=33
chi-sq: 992.2
motif: ^...s..S..E
match-: 16 48.5%
match+: 17 51.5%
expect: 0.240
enrich: 66.7
nanovial B #clones=33
chi-sq: 688.9
motif: A.sla..G..F
match-: 17 51.5%
match+: 21 63.6%
expect: 0.400
enrich: 42.5
A2/M1₅₈tetramer⁺ CD8⁺ T cells
CDR3α
cells A #clones=23
chi-sq: 326.6
motif: vkDa.G
match-: 13 56.5%
match+: 13 56.5%
expect: 0.480
enrich: 27.1
CDR3β
cells B #clones=23
chi-sq: 870.2
motif: l.as.E..f
match-: 15 65.2%
match+: 15 65.2%
expect: 0.080
enrich: 187.5
E
Sorted cells
Tetramer 23
Nanovials 33
F
IFNγ secretion
low 9
medium 9
bottom 5
high 10
Clone ID
A
B
D
F
G
H
unshared
G
Low IFNγ
Medium IFNγ
High IFNγ
Mean fluorescence intensity IFNγ-APC
25000
20000
15000
10000
5000
0
Clone ID
N B O G B A A F D P A A B A B G E B A M C A L B F E K H C J A I C D

Supplementary Figure 3. Comparable TCRαβ sequences among different nanovial- and tetramer-bound A2/M1$_{58}$-specific CD8^{+} T cells.

(A) Workflow for single-cell index sorting and TCRαβ multiplex RT-PCR sequence analysis, created with BioRender. (B) Representative gating of index-sorted IFNγ^{+}CD8^{+} T cell-loaded nanovials and A2/M1$_{58}$ tetramer^{+}CD8^{+} T cells. (C) Pairing of TRAV and TRBV clonotypes of nanovial-bound IFNγ^{+}CD8^{+} T cells and A2/M1$_{58}$ tetramer^{+}CD8^{+} T cells illustrated by circos plots. Left arch segment colored by TRAV usage, right outer arch colored by TRBV usage. Connecting lines indicated TRAV-TRBV gene pairing and are colored based on their TRAV usage and segmented based on their CRD3α and CDR3β sequence. The thickness is proportional to the number of TCR clones with the respective pair. The number of sequences considered for each circos plot is shown at the right bottom. (D) Top-scoring CDR3α and CDR3β sequence motifs for nanovial-bound A2/M1$_{58}$-specific CD8^{+} T cells (top) and A2/M1$_{58}$ tetramers-bound CD8^{+} T cells (bottom). Logo plots depict the V- (left side) and J- (right side) gene frequencies with the CDR3 amino acid sequence in the middle with the full height (above) and scaled (below) by per-residue reparative entropy to background frequencies derived from TCRs with matching gene-segment composition to highlight motif positions under selection. The middle section indicates the inferred rearrangement structure by source region (light grey for V-region, dark grey for J, black for D and red for N-insertions) of the grouped receptors. Motif scores were determined by chi-squared, with values above 90 considered significant. (E) Circos plots of paired TCRαβ clonotypes shared between nanovial-bound IFNγ^{+}CD8^{+} T cells and A2/M1$_{58}$ tetramer-bound CD8^{+} T cells. Connecting lines indicate shared clonotypes, colors are representative of the clonotypes/clone IDs found in Supplementary Table 1. (F) Circos plots of paired TCRαβ clonotypes between IFNγ secretion levels within the nanovial-bound IFNγ^{+}CD8^{+} T cell population. Connecting lines indicate shared clonotypes between the secretion levels, colors are representative of the clonotypes/clone IDs found in Supplementary Table 1. (G) IFNγ-APC mean fluorescent intensity (MFI) of index-sorted nanovial-bound A2/M1$_{58}$-specific CD8^{+} T-cells. Each dot represents a single cell, ordered by increasing IFNγ-APC MFI. The TCR clonotype ID of the cell is indicated below the graph and corresponds to the clone ID found in Supplementary Table 1. The black vertical lines denote the IFNγ-secretion levels Data obtained from one donor/experiment. Data obtained from one donor/experiment.
